# Risk of preterm birth, small for gestational age at birth, and stillbirth after covid-19 vaccination during pregnancy: population based retrospective cohort study

**DOI:** 10.1136/bmj-2022-071416

**Published:** 2022-08-17

**Authors:** Deshayne B Fell, Sheryll Dimanlig-Cruz, Annette K Regan, Siri E Håberg, Christopher A Gravel, Laura Oakley, Gillian D Alton, Eszter Török, Tavleen Dhinsa, Prakesh S Shah, Kumanan Wilson, Ann E Sprague, Darine El-Chaâr, Mark C Walker, Jon Barrett, Nannette Okun, Sarah A Buchan, Jeffrey C Kwong, Sarah E Wilson, Sandra I Dunn, Shannon E MacDonald, Shelley D Dougan

**Affiliations:** 1 Children’s Hospital of Eastern Ontario (CHEO) Research Institute, Ottawa, ON, Canada; 2 School of Epidemiology and Public Health, University of Ottawa, Ottawa, ON, Canada; 3 Better Outcomes Registry and Network (BORN) Ontario, Ottawa, ON, Canada; 4 School of Nursing and Health Professions, University of San Francisco, San Francisco, CA, USA; 5 Department of Epidemiology, UCLA Fielding School of Public Health, Los Angeles, CA, USA; 6 Centre for Fertility and Health, Norwegian Institute of Public Health, Oslo, Norway; 7 Department of Epidemiology, Biostatistics, and Occupational Health, McGill University, Montreal, QC, Canada; 8 Department of Epidemiology and Population Health, London School of Hygiene and Tropical Medicine, London, UK; 9 Department of Paediatrics, Mount Sinai Hospital, Toronto, ON, Canada; 10 Department of Paediatrics, University of Toronto, Toronto, ON, Canada; 11 Maternal-infant Care Research Centre, Department of Paediatrics, Mount Sinai Hospital, Toronto, ON, Canada; 12 Institute of Health Policy, Management, and Evaluation, University of Toronto, Toronto, ON, Canada; 13 Clinical Epidemiology Program, Ottawa Hospital Research Institute, Ottawa, ON, Canada; 14 Department of Medicine, University of Ottawa, Ottawa, ON, Canada; 15 Bruyère Research Institute, Ottawa, ON, Canada; 16 Department of Obstetrics and Gynaecology, University of Ottawa, Ottawa, ON, Canada; 17 Department of Obstetrics and Gynaecology, McMaster University, Hamilton, ON, Canada; 18 Department of Obstetrics and Gynaecology, University of Toronto, Toronto, ON, Canada; 19 Public Health Ontario, Toronto, ON, Canada; 20 Dalla Lana School of Public Health, University of Toronto, Toronto, ON, Canada; 21 ICES, Toronto, ON, Canada; 22 Department of Family and Community Medicine, University of Toronto, Toronto, ON, Canada; 23 School of Nursing, University of Ottawa, Ottawa, ON, Canada; 24 Faculty of Nursing, University of Alberta, Edmonton, AB, Canada; 25 School of Public Health, University of Alberta, Edmonton, AB, Canada; 26 Department of Paediatrics, University of Calgary, Calgary, AB, Canada

## Abstract

**Objective:**

To assess the risk of preterm birth, small for gestational age at birth, and stillbirth after covid-19 vaccination during pregnancy.

**Design:**

Population based retrospective cohort study.

**Setting:**

Ontario, Canada, 1 May to 31 December 2021.

**Participants:**

All liveborn and stillborn infants from pregnancies conceived at least 42 weeks before the end of the study period and with gestational age ≥20 weeks or birth weight ≥500 g.

**Main outcome measures:**

Using Cox regression, hazard ratios and 95% confidence intervals were estimated for preterm birth before 37 weeks (overall and spontaneous preterm birth), very preterm birth (<32 weeks), small for gestational age at birth (<10th centile), and stillbirth. Vaccination against covid-19 was treated as a time varying exposure in the outcome specific risk window, and propensity score weighting was used to adjust hazard ratios for potential confounding.

**Results:**

Among 85 162 births, 43 099 (50.6%) occurred in individuals who received one dose or more of a covid-19 vaccine during pregnancy—42 979 (99.7%) received an mRNA vaccine. Vaccination during pregnancy was not associated with any increased risk of overall preterm birth (6.5% among vaccinated *v* 6.9% among unvaccinated; adjusted hazard ratio 1.02, 95% confidence interval 0.96 to 1.08), spontaneous preterm birth (3.7% *v* 4.4%; 0.96, 0.90 to 1.03), or very preterm birth (0.59% *v* 0.89%; 0.80, 0.67 to 0.95). No increase was found in risk of small for gestational age at birth (9.1% *v* 9.2%; 0.98, 0.93 to 1.03) or stillbirth (0.25% *v* 0.44%; 0.65, 0.51 to 0.84). Findings were similar by trimester of vaccination, mRNA vaccine product, and number of doses received during pregnancy.

**Conclusion:**

The findings suggest that vaccination against covid-19 during pregnancy is not associated with a higher risk of preterm birth, small for gestational age at birth, or stillbirth.

## Introduction

Infection with SARS-CoV-2 during pregnancy has been associated with higher risks of admission to hospital, admission to an intensive care unit, and death for pregnant individuals.^1^ ^2^ ^3^ Furthermore, SARS-CoV-2 infection has been associated with a higher risk of preterm birth,^3^ ^4^ ^5^ fetal growth restriction,^4^ postpartum haemorrhage,^4^ and stillbirth.^6^ Many countries recommend covid-19 vaccination during pregnancy,^7^ which has been shown to be effective against covid-19 in pregnant individuals^8^ as well as their newborns^9^ ^10^; however, vaccine coverage among pregnant individuals remains lower than among women of reproductive age.^11^ ^12^

Safety concerns about covid-19 vaccination during pregnancy remains a potential obstacle to improving coverage. As of July 2022, results published from epidemiological studies are reassuring—two case-control studies of covid-19 vaccination in early pregnancy found no association with spontaneous abortion.^13^ ^14^ Our recent study of pregnancies to 30 September 2021, including 22 660 individuals vaccinated during the second or third trimester, did not show any association with adverse peripartum outcomes such as postpartum haemorrhage or low Apgar scores.^15^ However, fewer studies have examined risk of adverse birth outcomes associated with prenatal covid-19 vaccination. A population based birth registry study from Sweden and Norway found no increased risks of preterm birth or small for gestational age at birth; importantly, stillbirth also was not associated with covid-19 vaccination in the study.^16^ Two cohort studies including pregnant individuals insured through large health maintenance organisations observed no associations with preterm birth or small for gestational age at birth.^17^ ^18^ In a large, province-wide population, we evaluated whether covid-19 vaccination during pregnancy was associated with risk of preterm birth (including spontaneous preterm birth and very preterm birth), small for gestational age at birth, or stillbirth.

## Methods

We followed guidance for conducting studies of covid-19 vaccination during pregnancy^19^ and reporting observational studies.^20^

### Study design, population, and data sources

We conducted a population based retrospective cohort study in Ontario, Canada, which provides publicly funded healthcare to all residents, including services relating to prenatal and obstetrical care. The provincial birth registry (Better Outcomes Registry and Network (BORN Ontario))^21^ was used to identify the study population—after extracting records for completed pregnancies between 1 May and 31 December 2021, we extracted the corresponding births to create distinct records for each live birth and stillbirth, including multi-fetal pregnancies. We excluded births to non-Ontario residents and births from pregnancies conceived less than 42 weeks before the end of the study (ie, last menstrual period date after 10 March 2021) to avoid cohort truncation bias caused by overrepresentation of preterm births close to the end of the study period.^22^ We also excluded any records with gestational age <20 weeks and birth weight <500 g, or following pregnancy termination, as these events are not systematically collected in the registry (see supplementary table 1 for additional details of data sources).^21^

The birth registry receives records for all liveborn infants and stillborn infants ≥20 weeks’ gestation or with birth weight ≥500 g from hospitals, birth centres, and midwifery practice groups (home births) across Ontario.^21^ Maternal personal characteristics, health behaviours, pre-existing health conditions, pregnancy history, obstetric complications, interventions, and birth outcomes are collected from medical records, clinical forms, and patient interview and were shown to be of high quality in a validation study.^23^ Unique health card numbers (available for 96.9% of the study population) were used to deterministically link birth records to the COVaxON database, which captures all covid-19 immunisations in the province. Since 1 March 2020, information on individuals with confirmed covid-19 during pregnancy has been captured from two sources: a voluntary hospital based or midwifery practice group based case report form submitted directly to the birth registry, and through deterministic linkage with Ontario’s database containing all individuals with polymerase chain reaction (PCR) confirmed covid-19 reported to public health (Public Health Case and Contact Management Solution).^24^ During the study period, free PCR testing was widely available and recommended for anyone with symptoms of covid-19 or those in close contact with an individual with confirmed covid-19, or both. Finally, maternal residential postal code was used to link to area based socioeconomic data from Statistics Canada’s 2016 census and the Ontario Marginalization Index.^25^

### Outcome measures

#### Exposure variables

Ontario’s covid-19 vaccination programme began on 14 December 2020,^26^ and pregnant individuals were designated as a priority population for vaccination on 23 April 2021.^27^ Owing to a limited supply of covid-19 vaccines in Canada at that time, eligibility had not yet expanded to the general adult population; moreover, for several months during the spring of 2021, Canada used an extended dose interval of up to four months between the first and second vaccine dose.^28^ On 18 May 2021, all people older than 18 years became eligible to receive a covid-19 vaccine, and on 15 December 2021, all people older than 18 years, including pregnant people, became eligible to receive a covid-19 booster dose.^29^ From COVaxON, we identified vaccinations received between the estimated date of conception up to one day before birth. We treated vaccination as a time varying exposure within outcome specific risk windows—in the main analyses of preterm birth outcomes and stillbirth, ongoing pregnancies changed status from unvaccinated to vaccinated on the day that dose 1 was received in the risk window (see supplementary fig 1). For the assessment of small for gestational age at birth, we lagged the date of dose 1 by 14 days, since any potential effect of vaccination on fetal growth would not happen acutely.^16^ In subgroup analyses assessing the number of doses, an additional change in exposure status occurred if dose 2 was received in the risk window. Gestational timing of all doses received during pregnancy was classified as first trimester (pregnancy day 14 to 13 weeks+6 days), second trimester (14 weeks+0 days to 27 weeks+6 days), or third trimester (28 weeks+0 days to end of follow-up). Gestational age is recorded in the birth registry, and most pregnancy dating in Ontario is based on early ultrasound assessment.

#### Outcome variables

We defined preterm birth and very preterm birth as a live birth before 37 and 32 completed weeks of gestation, respectively. Preterm birth subtype was considered spontaneous if it occurred after spontaneous onset of labour or preterm premature rupture of membranes.^30^ Small for gestational age at birth was defined as a singleton live born infant below the 10th centile of the sex specific birth weight for gestational age distribution, based on a Canadian reference standard.^31^ Stillbirth was defined as an antepartum or intrapartum fetal death at ≥20 weeks, with the gestational timing of the event based on the date of birth (information on the exact timing of fetal death was not available). The end of the outcome specific risk windows were 36 weeks+6 days of gestation for preterm birth (pregnancy day 258), 31 weeks+6 days for very preterm birth (pregnancy day 223), and end of pregnancy for small for gestational age infants and stillbirths.

### Covariates

We adjusted for a range of covariates potentially associated with study outcomes or covid-19 vaccination, or both, using propensity score methods: maternal age at delivery (years), prepregnancy body mass index ≥30 (*v* <30), self-reported smoking status (yes or no) or substance use during pregnancy (yes or no), public health unit region (seven regions), pre-existing maternal health conditions (composite of asthma, chronic hypertension, diabetes, heart disease, thyroid disease; yes or no), parity (number of previous live births and stillbirths), multi-fetal pregnancy (yes or no), rural or urban residence, neighbourhood income fifths, neighbourhood marginalisation fifths (four dimensions: residential instability, material deprivation, dependency, ethnic concentration), calendar week of conception (categorical), and first visit for prenatal care in the first trimester (yes or no). Supplementary table 2 provides additional details on outcomes and covariates.

### Statistical analysis

We used discrete time survival analysis to account for the time dependent nature of vaccination status and study outcomes. Each pregnancy contributed gestational time in days starting on the estimated date of conception (pregnancy day 14); ongoing pregnancies on 1 May (start of the study) started contributing time from that point (see supplementary fig 1), and follow-up continued until either the event or censoring at the end of the outcome specific risk window.

We used extended Cox proportional hazards regression models with gestational age in days as the time scale, and robust sandwich variance estimation to account for statistical dependence across repeated observations as a result of changes in vaccination status, which was treated as time varying. We estimated hazard ratios with 95% confidence intervals and used inverse probability of treatment weights to generate adjusted hazard ratios.^32^ The weights were derived from a propensity score representing the predicted probability of having received one dose or more of a covid-19 vaccine during pregnancy—for vaccine exposed births, the weight was computed as the inverse of the propensity score, and for unexposed births, the inverse of 1 minus the propensity score. To account for any extreme weights, we stabilised the weights to the entire population and trimmed the values to the 0.01st to 99.99th centiles.^32^ Missing covariate values were assumed to be missing at random, and we imputed missing values before generating propensity scores using a fully conditional specification (MI procedure in SAS Version 9.4, SAS Institute, Cary, NC).^33^ The percentage of missing data for any individual covariate included in propensity scores was low (range 0-3.5%), with the exception of body mass index, which had 10.2% missing. Across all the covariates included in the propensity scores, 15.4% of records had missing information for one or more covariates. Weighted Cox regression models from which we derived adjusted results were fitted on each of the five imputed datasets, and the results were combined using the MIANALYZE procedure (SAS Institute). As the distribution of maternal age after weighting the study population with the stabilised inverse probability of treatment weights remained imbalanced across the two exposure groups (as indicated by a standardised difference >0.1^34^), all weighted models additionally included continuous maternal age.

In primary analyses, we evaluated vaccination status as receipt of one dose or more of a covid-19 vaccine in the outcome specific risk window. We performed subgroup analyses for preterm birth and small for gestational age at birth to evaluate trimester specific associations according to the number of doses (treating each dose as time varying), mRNA vaccine product for dose 1, and vaccine product combinations for those who received two mRNA doses; the number of events for the other outcomes was insufficient to conduct these subgroup analyses. In sensitivity analyses, we added covid-19 during pregnancy as a time varying covariate to the model and, separately, excluded individuals with a history of covid-19 during pregnancy. To assess for potential residual confounding, we stratified the original models by neighbourhood income (two highest and three lowest fifths) and limited the unvaccinated group to those who received their first vaccine dose after pregnancy, because in an earlier study of this population their baseline characteristics were shown to be more similar to individuals vaccinated during pregnancy than to those never vaccinated at any time.^15^ We also repeated the original analyses limited to singleton births.

### Patient and public involvement

Owing to covid-19 related resource and time constraints, it was not feasible to directly involve patients in the design, conduct, reporting, or writing of our study. Although no patients were directly involved, the study team included several obstetrical care providers who were involved from the outset of planning the study and brought forward their experiences from patient interactions related to covid-19 vaccination during pregnancy. These were taken into consideration in planning this research and its dissemination to ensure relevancy for a broad group of knowledge users, including pregnant people.

## Results

**Fig 1:**
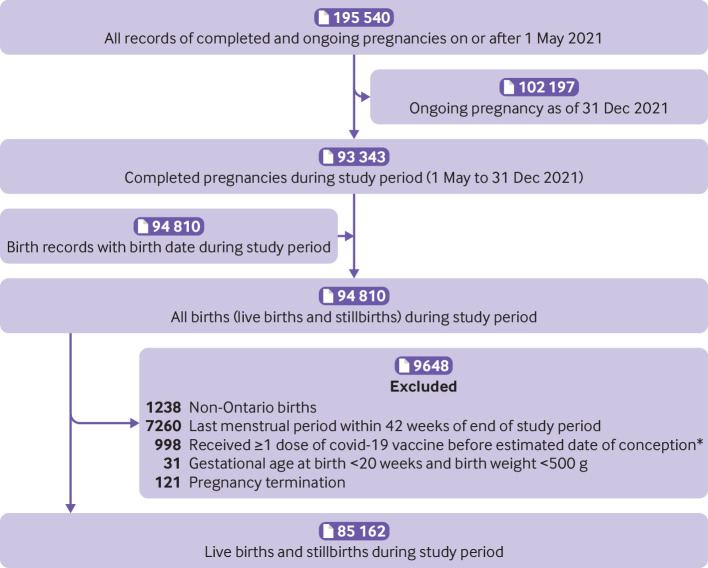
Study flow diagram. *Includes 366 who received a dose between the last menstrual period and estimated date of conception

After exclusions, 85 162 live births and stillbirths occurred during the study period (fig 1); of these, 43 099 (50.6%) were related to individuals who received one dose or more of a covid-19 vaccine during pregnancy. Those vaccinated during pregnancy were more likely to be ≥30 years of age, nulliparous, and live in the highest income neighbourhoods; they were less likely to be smokers, report substance use during pregnancy, or live in a rural setting (table 1 and table 2). A total of 3328 births (3.9%) were to individuals who had covid-19 during pregnancy. The proportion with covid-19 during pregnancy was lower in the vaccinated group than unvaccinated group (2.9% *v* 4.9%; table 2). However, most of the covid-19 episodes in the vaccinated group (1056/1247; 84.6%) preceded dose 1 by a median of 10.1 weeks (interquartile range 5.3-17.6 weeks).

**Table 1:** Personal characteristics of study population overall and by covid-19 vaccination status during pregnancy. Values are numbers (percentages) unless stated otherwise

Characteristics	Unweighted					Stabilised inverse probability of treatment weighted		
	Live births and stillbirths (n=85 162)*	≥1 vaccine dose during pregnancy (n=43 099)*	No vaccine during pregnancy (n=42 063)*	Standardised difference†		≥1 vaccine dose during pregnancy* (%)	No vaccine during pregnancy* (%)	Standardised difference†
**Maternal age (years)**								
<25	6877 (8.1)	1836 (4.3)	5041 (12.0)	0.29		6.7	9.0	0.08
25-29	20 338 (23.9)	8652 (20.1)	11 686 (27.8)	0.18		23.3	24.3	0.03
30-34	34 350 (40.3)	19 127 (44.4)	15 223 (36.2)	0.17		43.3	36.9	0.13
35-39	19 405 (22.8)	11 287 (26.2)	8118 (19.3)	0.16		22.7	22.9	0.00
≥40	4192 (4.9)	2197 (5.1)	1995 (4.7)	0.02		4.0	6.9	0.13
Mean (SD) age	32.1 (4.9)	32.9 (4.4)	31.3 (5.3)	0.05		32.1 (4.6)	32.2 (5.3)	0.01
**Estimated date of conception**								
Before Sept 2020	7059 (8.3)	2376 (5.5)	4683 (11.1)	0.20		6.7	9.1	0.09
Sept-Oct 2020	24 396 (28.6)	11 309 (26.2)	13 087 (31.1)	0.11		29.8	27.3	0.05
Nov-Dec 2020	24 470 (28.7)	12 586 (29.2)	11 884 (28.3)	0.02		29.3	28.3	0.02
Jan-Feb 2021	21 922 (25.7)	12 538 (29.1)	9384 (22.3)	0.16		26.0	25.7	0.01
After Feb 2021	7315 (8.6)	4290 (10.0)	3025 (7.2)	0.10		8.3	9.5	0.04
**Parity**								
0 (nulliparous)	36 932 (43.4)	19 689 (45.7)	17 243 (41.0)	0.09		43.6	43.7	0.00
≥1 (multiparous)	47 835 (56.2)	23 176 (53.8)	24 659 (58.6)	0.10		56.4	56.3	0.00
Missing‡	395 (0.5)	234 (0.5)	161 (0.4)	0.02				
**Multiple birth**								
Yes	2416 (2.8)	1235 (2.9)	1181 (2.8)	0.00		2.8	2.9	0.00
No	82 746 (97.2)	41 864 (97.1)	40 882 (97.2)	0.00		97.2	97.1	0.00
**Pre-existing medical condition§**								
Yes	9474 (11.1)	5253 (12.2)	4221 (10.0)	0.07		11.2	11.2	0.00
Thyroid disease	5018 (5.9)	2957 (6.9)	2061 (4.9)	0.08		6.1	5.8	0.01
Asthma	3279 (3.9)	1683 (3.9)	1596 (3.8)	0.01		3.7	3.9	0.01
Diabetes	910 (1.1)	483 (1.1)	427 (1.0)	0.01		1.1	1.1	0.00
Chronic hypertension	761 (0.9)	414 (1.0)	347 (0.8)	0.01		0.9	0.9	0.01
Heart disease	100 (0.1)	56 (0.1)	44 (0.1)	0.01		0.1	0.1	0.00
**Smoked during pregnancy**								
No	78 103 (91.7)	40 566 (94.1)	37 537 (89.2)	0.18		93.4	93.7	0.01
Yes	5168 (6.1)	1461 (3.4)	3707 (8.8)	0.23		6.6	6.3	0.01
Missing‡	1891 (2.2)	1072 (2.5)	819 (1.9)	0.04				
**Substance use during pregnancy**¶								
No	78 346 (92.0)	40 282 (93.5)	38 064 (90.5)	0.11		94.7	94.9	0.01
Yes	3958 (4.6)	1264 (2.9)	2694 (6.4)	0.17		5.3	5.1	0.01
Missing‡	2858 (3.4)	1553 (3.6)	1305 (3.1)	0.03				
**Prepregnancy BMI**								
<30	60 358 (70.9)	31 024 (72.0)	29 334 (69.7)	0.05		78.8	78.9	0.00
≥30 (obese)	16 100 (18.9)	7972 (18.5)	8128 (19.3)	0.02		21.2	21.1	0.00
Missing‡	8704 (10.2)	4103 (9.5)	4601 (10.9)	0.05				
**Neighbourhood median family income fifth**								
First (lowest)	17 118 (20.1)	7081 (16.4)	10 037 (23.9)	0.19		20.5	20.3	0.00
Second	17 037 (20.0)	8144 (18.9)	8893 (21.1)	0.06		20.1	20.2	0.00
Third	17 973 (21.1)	9154 (21.2)	8819 (21.0)	0.01		21.2	21.3	0.00
Fourth	17 994 (21.1)	9989 (23.2)	8005 (19.0)	0.10		21.3	21.3	0.00
Fifth (highest)	14 271 (16.8)	8525 (19.8)	5746 (13.7)	0.16		16.9	16.9	0.00
Missing‡	769 (0.9)	206 (0.5)	563 (1.3)	0.09				
Rural residence**								
No	73 112 (85.9)	37 896 (87.9)	35 216 (83.7)	0.12		85.6	85.8	0.01
Yes	12 050 (14.1)	5203 (12.1)	6847 (16.3)	0.12		14.4	14.2	0.01
**Public health unit region of residence**								
Central east	23 780 (27.9)	11 883 (27.6)	11 897 (28.3)	0.02		28.4	28.4	0.00
Central west	18 006 (21.1)	8824 (20.5)	9182 (21.8)	0.03		21.2	21.3	0.00
Greater Toronto region	16 620 (19.5)	9508 (22.1)	7112 (16.9)	0.13		19.6	19.7	0.00
Eastern	11 224 (13.2)	6347 (14.7)	4877 (11.6)	0.09		13.2	13.2	0.00
South west	10 494 (12.3)	4478 (10.4)	6016 (14.3)	0.12		12.2	12.3	0.00
North east	2889 (3.4)	1229 (2.9)	1660 (3.9)	0.06		3.5	3.4	0.00
North west	1390 (1.6)	631 (1.5)	759 (1.8)	0.03		1.8	1.7	0.01
Missing‡	759 (0.9)	199 (0.5)	560 (1.3)	0.09				
**Residential instability fifth**								
First (least)	17 007 (20.0)	8818 (20.5)	8189 (19.5)	0.02		20.1	20.2	0.00
Second	15 810 (18.6)	8181 (19.0)	7629 (18.1)	0.02		18.8	18.8	0.00
Third	16 032 (18.8)	8167 (18.9)	7865 (18.7)	0.01		19.1	19.1	0.00
Fourth	15 528 (18.2)	7713 (17.9)	7815 (18.6)	0.02		18.6	18.5	0.00
Fifth (most)	19 286 (22.6)	9743 (22.6)	9543 (22.7)	0.00		23.4	23.4	0.00
Missing‡	1499 (1.8)	477 (1.1)	1022 (2.4)	0.10				
**Material deprivation fifth**								
First (least)	19 351 (22.7)	11 855 (27.5)	7496 (17.8)	0.23		23.0	23.1	0.00
Second	17 132 (20.1)	9481 (22.0)	7651 (18.2)	0.10		20.3	20.4	0.00
Third	15 461 (18.2)	7776 (18.0)	7685 (18.3)	0.01		18.3	18.3	0.00
Fourth	15 279 (17.9)	7145 (16.6)	8134 (19.3)	0.07		18.3	18.2	0.00
Fifth (most)	16 440 (19.3)	6365 (14.8)	10 075 (24.0)	0.23		20.1	20.0	0.00
Missing‡	1499 (1.8)	477 (1.1)	1022 (2.4)	0.10				
**Dependency fifth**								
First (least)	27 572 (32.4)	14 761 (34.2)	12 811 (30.5)	0.08		32.8	32.8	0.00
Second	17 547 (20.6)	8958 (20.8)	8589 (20.4)	0.01		20.8	20.9	0.00
Third	14 076 (16.5)	7104 (16.5)	6972 (16.6)	0.00		16.9	16.9	0.00
Fourth	12 749 (15.0)	6211 (14.4)	6538 (15.5)	0.03		15.2	15.2	0.00
Fifth (most)	11 719 (13.8)	5588 (13.0)	6131 (14.6)	0.05		14.3	14.2	0.00
Missing‡	1499 (1.8)	477 (1.1)	1022 (2.4)	0.10				
**Ethnic concentration fifth**								
First (lowest)	11 634 (13.7)	5357 (12.4)	6277 (14.9)	0.07		14.4	14.2	0.01
Second	13 640 (16.0)	6878 (16.0)	6762 (16.1)	0.00		16.3	16.3	0.00
Third	15 114 (17.7)	8251 (19.1)	6863 (16.3)	0.07		17.8	17.9	0.00
Fourth	18 378 (21.6)	9898 (23.0)	8480 (20.2)	0.07		21.8	21.9	0.00
Fifth (highest)	24 897 (29.2)	12 238 (28.4)	12 659 (30.1)	0.04		29.6	29.7	0.00
Missing‡	1499 (1.8)	477 (1.1)	1022 (2.4)	0.10				

BMI=body mass index; SD=standard deviation.

* Column percentages in the weighted study population were based on imputation dataset 1.

† Absolute standardised difference comparing those who received one dose or more of a covid-19 vaccine during pregnancy and those who were not vaccinated during pregnancy; standardised difference >0.10 indicates an imbalance in the distribution of the baseline characteristic between these two vaccination groups.

‡ Missing data are not shown in the weighted study population as multiple imputation was used before deriving inverse probability of treatment weights.

§ Composite of asthma, chronic hypertension, diabetes, heart disease, thyroid disease. Sum of individual conditions does not equal the total number of individuals with any individual condition, as categories were not mutually exclusive.

¶ Self-reported cannabis, opioid, or alcohol use during pregnancy.

** Because of small cell counts (<6) for missing values in the vaccinated group, missing has been combined with the “no” category.

**Table 2:** Pregnancy characteristics of study population overall and by covid-19 vaccination status during pregnancy. Values are numbers (percentages) unless stated otherwise

Characteristics	Unweighted					Stabilised inverse probability of treatment weighted		
	Live births and stillbirths (n=85 162)*	≥ 1 vaccine dose during pregnancy (n=43 099)*	No vaccine during pregnancy (n=42 063)*	Standardised difference†		≥1 vaccine dose during pregnancy * (%)	No vaccine during pregnancy * (%)	Standardised difference†
**Covid-19 during pregnancy‡**								
Yes	3328 (3.9)	1247 (2.9)	2081 (4.9)	0.11		3.1	4.8	0.09
No	81 834 (96.1)	41 852 (97.1)	39 982 (95.1)	0.11		96.9	95.2	0.09
**First prenatal care visit in first trimester**								
Yes	77 440 (90.9)	39 623 (91.9)	37 817 (89.9)	0.07		94.1	94.2	0.00
No	4755 (5.6)	1852 (4.3)	2903 (6.9)	0.11		5.9	5.8	0.00
Missing§	2967 (3.5)	1624 (3.8)	1343 (3.2)	0.03				
**Birth location**								
Home	2460 (2.9)	845 (2.0)	1615 (3.8)	0.11		2.0	3.7	0.10
Hospital	82 103 (96.4)	41 994 (97.4)	40 109 (95.4)	0.11		97.4	95.5	0.10
Birth centre	382 (0.4)	197 (0.5)	185 (0.4)	0.00		0.4	0.5	0.02
Clinic (midwifery)	153 (0.2)	44 (0.1)	109 (0.3)	0.04		0.1	0.2	0.02
Other Ontario location	64 (0.1)	19 (0.0)	45 (0.1)	0.02		0.1	0.1	0.01
**Healthcare provider**								
Midwives	9892 (11.6)	4499 (10.4)	5393 (12.8)	0.07		10.4	12.7	0.07
CNS, NP, registered nurse	590 (0.7)	240 (0.6)	350 (0.8)	0.03		0.6	0.8	0.02
Family doctor	4939 (5.8)	2547 (5.9)	2392 (5.7)	0.01		6.5	5.3	0.05
Obstetrician	61 534 (72.3)	31 399 (72.9)	30 135 (71.6)	0.03		72.7	72.7	0.00
Other healthcare provider, resident, surgeon	7422 (8.7)	4023 (9.3)	3399 (8.1)	0.04		9.5	8.2	0.05
Unattended	263 (0.3)	85 (0.2)	178 (0.4)	0.04		0.2	0.4	0.03
Missing§	522 (0.6)	306 (0.7)	216 (0.5)	0.03				

CNS=clinical nurse specialist; NP=nurse practitioner.

* Column percentages in the weighted study population were based on imputation dataset 1.

† Absolute standardised difference comparing those who received one dose or more of a covid-19 vaccine during pregnancy and those who were not vaccinated during pregnancy; standardised difference >0.10 indicates an imbalance in the distribution of the baseline characteristic between these two vaccination groups.

‡ Laboratory confirmed covid-19 between estimated date of conception up to one day before date of birth (the specimen collection date, which was a proxy for date of infection, was lagged by two days).

§ Missing data are not shown in the weighted study population as multiple imputation was used before deriving inverse probability of treatment weights.

**Fig 2:**
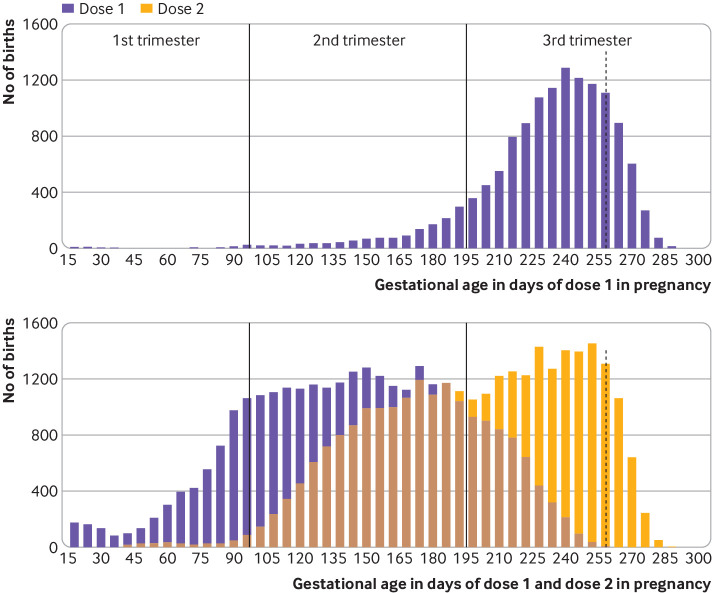
Gestational timing of covid-19 vaccine doses received during pregnancy among 43 099 pregnant individuals in Ontario, Canada. (Top panel) Gestational age in days when dose 1 was received, among births to individuals who received only one vaccine dose during pregnancy (n=13 416). (Bottom panel) Gestational age in days when dose 1 and dose 2 were received, among births to individuals who received only two doses during pregnancy (n=29 650). Data for individuals who received three doses during pregnancy (n=33) are not shown. Vertical dotted line represents the end of the risk window for preterm birth (pregnancy day 258)

Among 43 099 individuals who were vaccinated during pregnancy, 13 416 (31.1%) received one vaccine dose, 29 650 (68.8%) received two doses, and 33 (0.1%) received three doses (table 3). Overall, 12.1% (n=5213) received dose 1 during the first trimester, 48.1% (n=20 715) during the second trimester, and 39.8% (n=17 171) during the third trimester. The median gestational age at dose 1 was 25 (interquartile range 18-32) weeks—later, when only one dose was received during pregnancy (median 34 (interquartile range 31-36) weeks; n=13 416) and earlier when two doses were received during pregnancy (median 21 (interquartile range 16-26) weeks; n=29 650) (fig 2). Overall, 80.1% (n=34 526) of dose 1 administrations during pregnancy were BNT162b2 (Comirnaty; Pfizer-BioNTech) and 19.6% (n=8453) were mRNA-1273 (Spikevax; Moderna); <1% (0.3%; n=120) were another vaccine product (Vaxzevria; Oxford-AstraZeneca: n=101; other: n=19).

**Table 3:** Vaccination characteristics among 43 099 pregnant individuals who received one dose or more of a covid-19 vaccine during pregnancy. Values are numbers (percentages) unless stated otherwise

Characteristics	≥1 vaccine dose during pregnancy (n=43 099)
**No of doses received during pregnancy**	
One dose	13 416 (31.1)
Two doses	29 650 (68.8)
Three doses	33 (0.1)
**No of doses received during and/or after pregnancy**	
One dose during pregnancy	422 (1.0)
One dose during, one dose after, pregnancy	5555 (12.9)
One dose during, two doses after, pregnancy	7439 (17.3)
Two doses during pregnancy	9646 (22.4)
Two doses during, one dose after, pregnancy	20 004 (46.4)
Three doses during pregnancy	33 (0.1)
**Trimester of vaccine dose 1 (overall)**	
First	5213 (12.1)
Second	20 715 (48.1)
Third	17 171 (39.8)
Median (IQR) gestational age (days)	176 (126-222)
Median (IQR) gestational age (weeks)	25.1 (18.0-31.7)
Trimester of vaccine dose 1 (only one dose during pregnancy; n=13 416):	
First	93 (0.7)
Second	1409 (10.5)
Third	11 914 (88.8)
Median (IQR) gestational age (days)	237 (217-253)
Median (IQR) gestational age (weeks)	33.9 (31.0-36.1)
Trimester of vaccine dose 1 (only two doses during pregnancy; (n=29 650):	
First	5091 (17.2)
Second	19 302 (65.1)
Third	5257 (17.7)
Median (IQR) gestational age (days)	147 (110-184)
Median (IQR) gestational age (weeks)	21.1 (15.7-26.3)
**Trimester of vaccine dose 2 (only two doses during pregnancy; n=29 650)**	
First	301 (1.0)
Second	13 019 (43.9)
Third	16 330 (55.1)
Median (IQR) gestational age (days)	204 (165-239)
Median (IQR) gestational age (weeks)	29.1 (23.6-34.1)
**Vaccine product received for dose 1**	
BNT162b2 (Pfizer-BioNTech)	34 526 (80.1)
mRNA-1273 (Moderna)	8453 (19.6)
Other*	120 (0.3)
**Vaccine product for those who received two doses of mRNA vaccine during pregnancy (n=29 574)**	
BNT162b2+BNT162b2†	19 866 (67.2)
mRNA-1273+mRNA-1273†	5321 (18.0)
BNT162b2+mRNA-1273 or mRNA-1273+BNT162b2‡	4387 (14.8)

IQR=interquartile range.

* Includes non-mRNA covid-19 vaccines (eg, manufactured by Oxford-AstraZeneca and Johnson & Johnson-Janssen).

† Same type of mRNA vaccine for doses 1 and 2 (homologous mRNA series).

‡ Different type of mRNA vaccine for doses 1 and 2 (heterologous mRNA series).

Overall, 5719 (6.7%) preterm births occurred; 3450 (4.1%) were spontaneous preterm births (table 4). The cumulative incidence of overall preterm birth was 6.5% among those who were vaccinated during pregnancy and 6.9% among those who were unvaccinated during pregnancy. Vaccination was not associated with an increased risk of overall preterm birth (adjusted hazard ratio 1.02, 95% confidence interval 0.96 to 1.08), spontaneous preterm birth (0.96, 0.90 to 1.03), or very preterm birth (0.80, 0.67 to 0.95). In subgroup analyses, results for overall preterm birth were similar by trimester (adjusted hazard ratios: first trimester, 0.98, 95% confidence interval 0.87 to 1.10; second trimester 0.98, 0.91 to 1.04; third trimester 1.00, 0.92 to 1.08) and vaccine product for dose 1, as well as by number of doses received during pregnancy, and vaccine product combination for those who received both doses during pregnancy (see supplementary table 3). Subgroup analyses could not be conducted for spontaneous preterm birth or very preterm birth owing to the small number of events.

**Table 4:** Association between covid-19 vaccination during pregnancy and study outcomes

Outcomes	No vaccine during pregnancy (n=42 063)	≥1 vaccine dose during pregnancy* (n=43 099)
**Preterm birth <37 weeks†**		
No of participants	41 879	42 992
No (%) with outcome (rate/100 live births)	2907 (6.9)	2812 (6.5)
No of pregnancy days at risk‡	4 304 319	3 410 735
Unadjusted hazard ratio (95% CI)	1.00	0.97 (0.92 to 1.02)
Adjusted hazard ratio (95% CI)	1.00	1.02 (0.96 to 1.08)
**Spontaneous preterm birth <37 week†§**		
No of participants	41 879	42 992
No (%) with outcome (rate/100 live births)	1842 (4.4)	1608 (3.7)
No of pregnancy days at risk‡	4 304 319	3 410 735
Unadjusted hazard ratio (95% CI)	1.00	0.88 (0.82 to 0.94)
Adjusted hazard ratio (95% CI)	1.00	0.96 (0.90 to 1.03)
**Very preterm birth <32 weeks†**		
No of participants	41 879	42 992
No (%) with outcome (rate/100 live births)	374 (0.89)	252 (0.59)
No of pregnancy days at risk‡	3 081 508	2 190 543
Unadjusted hazard ratio (95% CI)	1.00	0.79 (0.67 to 0.93)
Adjusted hazard ratio (95% CI)	1.00	0.80 (0.67 to 0.95)
**Small for gestational age at birth¶**		
No of participants	40 280	41 333
No (%) with outcome (rate/100 singleton live births)	3722 (9.2)	3743 (9.1)
No of pregnancy days at risk‡	4 783 558	3 953 597
Unadjusted hazard ratio (95% CI)	1.00	0.96 (0.91 to 1.00)
Adjusted hazard ratio (95% CI)	1.00	0.98 (0.93 to 1.03)
**Stillbirth**		
No of participants	42 063	43 099
No (%) with outcome (rate/100 live births and stillbirths)	184 (0.44)	107 (0.25)
No of pregnancy days at risk‡	4 994 562	4 111 719
Unadjusted hazard ratio (95% CI)	1.00	0.65 (0.51 to 0.82)
Adjusted hazard ratio (95% CI)	1.00	0.65 (0.51 to 0.84)

CI=confidence interval.

* Vaccination was treated as a time varying exposure within outcome specific risk windows. Hazard ratios were adjusted using stabilised inverse probability of treatment weights, trimmed at 0.01st and 99.99th centiles. In addition to weights, maternal age (as a continuous variable) was added to the adjusted models as it remained imbalanced between the two groups after weighting.

† Among live births only.

‡ Number of days at risk in risk window. Preterm birth: 36 weeks+6 days of gestation (pregnancy day 258); very preterm birth: 31 weeks+6 days of gestation (pregnancy day 223); small for gestational age at birth and stillbirth: end of pregnancy. Since vaccination status was treated as a time varying variable, vaccinated individuals could have contributed both exposed and unexposed days at risk.

§ For spontaneous preterm birth, medically initiated preterm births were censored at delivery.

¶ Among singleton live births only. A total of 885 records were excluded from the analysis (34 records had a gestational age either below or above the values provided in the reference standard used to classify small for gestational age at birth, and 851 records had missing information on infant sex or birth weight, or both).

The cumulative incidence of small for gestational age at birth was 9.1% (n=3743) among births to individuals who received one dose or more of a covid-19 vaccine during pregnancy, and 9.2% (n=3722) among births to unvaccinated individuals. No association was observed between vaccination during pregnancy and small for gestational age at birth overall (adjusted hazard ratio 0.98, 0.93 to 1.03; table 4), or in subgroup analyses by trimester and vaccine product for dose 1 (see supplementary table 3). A small increased association was observed for only dose 1 administered during pregnancy (1.09, 1.01 to 1.16), which was not observed among individuals who received two doses during pregnancy (0.92, 0.87 to 0.97; see supplementary table 3). The cumulative incidence of stillbirth was 0.25% (n=107) among births to individuals who received one dose or more of a covid-19 vaccine during pregnancy, and 0.44% (n=184) among births to unvaccinated individuals. Vaccination was not associated with any increase in the risk of stillbirth (0.65, 0.51 to 0.84; table 4). The number of stillbirths was not large enough for subgroup analyses.

### Sensitivity analyses

When covid-19 during pregnancy was added to the model as a time varying exposure and when births to individuals with covid-19 during pregnancy were excluded, the results remained unchanged for all outcomes (see supplementary table 4). Estimates for most outcomes were similar across strata of neighbourhood income; although stratum specific estimates for individuals of higher income (fourth and fifth fifths) and lower income (first to third fifths) were different in magnitude for very preterm birth and for stillbirth, the direction of the stratum specific point estimates was consistent with that of the main analyses, and confidence intervals overlapped. When the comparison group was restricted to unvaccinated individuals who initiated their covid-19 vaccine series after pregnancy, the results for all outcomes remained unchanged. Analyses restricted to singleton births were similar for preterm birth outcomes; however, the estimate for stillbirth moved closer to the null value (adjusted hazard ratio 0.74, 0.57 to 0.96).

## Discussion

In this large population of more than 85 000 live births and stillbirths up to 31 December 2021, we found no evidence that vaccination during pregnancy with an mRNA covid-19 vaccine was associated with a higher risk of preterm birth, spontaneous preterm birth, very preterm birth, small for gestational age at birth, or stillbirth. These results—based on more than 43 000 fetuses exposed to at least one dose of mRNA covid-19 vaccine—did not differ by trimester of vaccination, number of doses received during pregnancy, or mRNA vaccine product.

### Comparison with other studies

Our findings for preterm birth and small for gestational age at birth are consistent with a recent Scandinavian birth registry based study of 157 521 births (103 409 from Sweden and 54 112 from Norway) to 12 January 2022, in which 18% of the births (n=28 506) were to individuals who were vaccinated during pregnancy.^16^ The adjusted estimates pooled across Sweden and Norway were consistent in magnitude and direction with our results for preterm birth (adjusted hazard ratio 0.98, 95% confidence interval 0.91 to 1.05) and small for gestational age at birth (0.97, 0.90 to 1.04).^16^ A US Vaccine Safety Datalink study of 46 079 singleton live births to 22 July 2021 (21.8% (n=10 064) of individuals received a covid-19 vaccine during pregnancy) reported adjusted hazard ratios for preterm birth (0.91, 95% confidence interval 0.82 to 1.01) and small for gestational age at birth (0.95, 0.87 to 1.03) that were similar to our estimates.^17^ An Israeli cohort study of 24 288 singleton live births to 30 September 2021 (68.7% (n=16 697) individuals received BNT162b2 during pregnancy)^18^ reported no increased risk of preterm birth (adjusted risk ratio 0.95, 95% confidence interval 0.83 to 1.10) or small for gestational age at birth (0.97, 0.87 to 1.08), and no differences in risk of infants being admitted to hospital or mortality up to 7 months of age.^18^ The Scandinavian study^16^ and the Vaccine Safety Datalink study^17^ both used a similar methodology as our study—treating vaccination during pregnancy as a time varying exposure—as has been recommended.^19^ Similar to our results, neither of these two studies^16^ ^17^ observed any patterns of concern when outcomes were assessed by number of vaccine doses, mRNA vaccine product, or trimester of vaccination.

We observed a reduction in stillbirth risk among vaccinated individuals (adjusted hazard ratio 0.65, 95% confidence interval 0.51 to 0.84), even after adjustment for a range of potential confounders through propensity score weighting. A reduced risk of stillbirth was also observed in the Scandinavian study, although the estimate was smaller in magnitude (pooled adjusted hazard ratio 0.86, 95% confidence interval 0.63 to 1.17).^16^ Collectively, the findings from these two studies are reassuring and are consistent with no increased risk of stillbirth after covid-19 vaccination during pregnancy. In contrast, covid-19 disease during pregnancy has been associated with an increased risk of stillbirth.^6^ Although fetal viraemia from SARS-CoV-2 transmission across the placenta is considered uncommon, it is biologically plausible that vaccination could protect against stillbirth through preventing SARS-CoV-2-associated placental damage^35^ or other immunological responses to clinical or subclinical infection. Nevertheless, we did not observe any difference in our findings in sensitivity analyses adjusting for confirmed covid-19 during pregnancy or after excluding individuals with covid-19 during pregnancy, suggesting it is unlikely to fully account for the suggested protective benefit of vaccination during pregnancy. Residual confounding by unmeasured characteristics or temporal issues could also have biased the estimate away from the null value.^19^ ^22^ Interestingly, a reduced stillbirth risk after influenza vaccination during pregnancy was also reported in some large cohort studies of 2009 pandemic A/H1N1 monovalent influenza vaccines and seasonal trivalent influenza vaccines—adjusted risk estimates for stillbirth ranged from 0.44 to 0.88 and several were statistically significant.^36^ Similar to influenza vaccination,^37^ vaccine derived maternal antibodies are known to cross the placenta after covid-19 vaccination during pregnancy,^38^ and emerging evidence supports protective benefits to newborns from covid-19 in the early months of life.^9^ ^10^

### Strengths and limitations of this study

Strengths of this study include the large birth population and number of individuals vaccinated during pregnancy. We used deterministically linked population based data sources within a publicly funded healthcare system; thus, identification of births and vaccinations was highly complete and minimised potential selection or exposure misclassification bias. As gestational age at birth is mostly based on early ultrasound assessment in Ontario, the accuracy of classifying gestational timing of vaccination was probably high. Although we adjusted for many potential confounders using a propensity score based approach, we cannot dismiss the possibility of residual confounding, particularly given the potential for healthy vaccinee bias in observational studies of vaccination.^19^ Moreover, despite using recommended methodological approaches for handling time dependent exposures and pregnancy outcomes,^19^ ^22^ we cannot rule out residual temporal confounding, particularly given the complex temporal dynamics of the pandemic and vaccination programme. We used a comprehensive strategy to identify cases of covid-19 during pregnancy, including the province-wide public health database for polymerase chain reaction confirmed covid-19; however, individuals who did not seek testing could be misclassified as not having had covid-19 during pregnancy. Pregnancies ending before 20 weeks’ gestation are not systematically captured in the birth registry and could not be evaluated; however, previous high quality case-control studies did not find any evidence of an increased risk of miscarriage associated with covid-19 vaccination during early pregnancy.^13^ ^14^ Despite the large sample size, our study might have been underpowered to rule out small associations for some rarer outcomes. During the study period, the proportion of vaccines administered in the first trimester was relatively low (12.1%). Moreover, we were unable to assess covid-19 vaccination before pregnancy or around the time of conception, as the time period covered by our study did not include sufficient numbers of these early vaccinations; evaluation of outcomes after vaccination in these time windows is planned for in future studies. We were unable to evaluate booster doses because pregnant Ontario residents were not eligible until December 2021, and most of these pregnancies are ongoing. Finally, we were limited to assessment of mRNA vaccine products, as use of other covid-19 vaccine types in the pregnant population in Canada has been limited.

### Conclusions

We did not find evidence of an increased risk of preterm birth, small for gestational age at birth, or stillbirth after covid-19 vaccination during any trimester of pregnancy in this large population based study including more than 43 000 births to individuals vaccinated during pregnancy. Our findings—along with extant evidence that vaccination during pregnancy is effective against covid-19 for pregnant individuals and their newborns, and that covid-19 during pregnancy is associated with increased risks of adverse maternal, fetal, and neonatal outcomes—can inform evidence based decision making about covid-19 vaccination during pregnancy. Future studies to assess similar outcomes after immunisation with non-mRNA covid-19 vaccine types during pregnancy should be a research priority.

**What is already known on this topic**

SARS-CoV-2 infection during pregnancy is associated with adverse maternal and birth outcomes
Pregnancy specific safety information about covid-19 vaccination is important for pregnant individuals, healthcare providers, and policy makers to guide decision making
Evidence from large comparative studies about pregnancy outcomes after covid-19 vaccination during pregnancy is limited

**What this study adds**

No association was found between immunisation with an mRNA covid-19 vaccine during pregnancy and increased risk of preterm birth, spontaneous preterm birth, very preterm birth, small for gestational age at birth, or stillbirth
These findings can help inform evidence based decision making about the risks and benefits of covid-19 vaccination during pregnancy

## Data Availability

The dataset from this study is held securely by BORN Ontario. Although the dataset cannot be made publicly available, the analytical code may be available on request.

## Web extra

Extra material supplied by authors

## Notes

Contributors: DBF, JCK, AES, DE-C, and SDD conceived the study. DBF, AKR, GDA, LO, CAG, and SEH developed the study design and analytical approach, in consultation with other project team members. GDA, TD, SD-C, and DBF linked the data sources. SD-C performed the statistical analyses, which were supervised by DBF. DBF drafted the initial version of the manuscript. All authors contributed to the interpretation of the findings and reviewed and edited the manuscript for intellectual content. All authors approved the final version of the manuscript and agreed to be accountable for all aspects of the work. DBF is the guarantor. The corresponding author attests that all listed authors meet authorship criteria and that no others meeting the criteria have been omitted.

The corresponding author (DBF) affirms that the manuscript is an honest, accurate, and transparent account of the study being reported; that no important aspects of the study have been omitted; and that any discrepancies from the study as originally planned (and, if relevant, registered) have been explained.

Dissemination to participants and related patient and public communities: All personal identifying information of study participants has been removed from the dataset; thus it is not possible to send the results of this study to participants. Given the rules under which the data are used for research purposes, individuals to whom the data relate are not permitted to be contacted. However, as part of a detailed knowledge translation plan, several provincial and national organisations have been engaged to assist with dissemination of the findings of this study to their diverse networks of public health units and practitioners, care providers, policy makers, and other stakeholders, including patients and the general public. The results of this study have also been shared with the public through provincial webinars and social media channels.

Provenance and peer review: Not commissioned; externally peer reviewed.
