# Supplementary material for: Risk of preterm birth, small for gestational age at birth, and stillbirth after covid-19 vaccination during pregnancy: population based retrospective cohort study
Source: BMJ. 2022 Aug 17;378:e071416. doi: 10.1136/bmj-2022-071416 (PMC9382031; doi:10.1136/bmj-2022-071416)
Supplement: Supplementary file 1 — Supplementary information: additional tables 1-4 [file feld071416.ww.pdf]

Supplementary Appendix

This appendix has been provided by the authors to give readers additional information about their work.

Supplement to: Risk of preterm birth, small for gestational age at birth, and stillbirth after covid-19 vaccination during pregnancy: population based retrospective cohort study

Contents

Supplementary Table 1. Description of data sources....2
Supplementary Table 2. Description of study variables from the BORN Ontario birth registry....4
Supplementary Figure 1. Study design....5
Supplementary Table 3. Subgroup analyses....7
Supplementary Table 4. Sensitivity analyses....9

Supplementary Table 1. Description of data sources

**BORN Information System**	Details about the BORN Information System (BIS) are available elsewhere.[1] Briefly, the BIS captures information on Ontario births ≥20 weeks' gestation or ≥500 grams (all hospital births and the ~2.8% of home births with a midwife[2]) from over 250 hospitals, birth centres, midwifery practice groups, and prenatal screening labs. Prenatal and maternity care are publicly[1] funded in the province.<br><br>Records for pregnant individuals who undergo prenatal screening (about 70% in Ontario[3]) are uploaded weekly to the BIS from hospital- and community-based labs and ultrasound clinics. Thus, screened pregnancies are identifiable in the BIS as early as 10 weeks' gestation; 97% of prenatal screening records are linked with other health care encounters pertaining to birth. Unscreened pregnancies typically become identifiable in the BIS only at the time of the birth.<br><br>When a pregnant individual presents to care around the time of birth, the BIS generates a "Labour and birth encounter" that captures information about labour and birth through to the first hour postpartum, regardless of birth setting (hospital, home, or birth centre). A separate "Birth-child encounter" documents information about each newborn (live births and stillbirths) through to the first hour postpartum. Postpartum encounters then capture clinical information about the mother and newborn(s) from the immediate postpartum period until discharge from hospital/birth centre. Unique identifiers (mother and newborn), assigned upon first record entry into the BIS, are used by the system to deterministically link all encounters through a robust automated algorithm. A system-generated signal indicates when each encounter record is complete.<br><br>The BIS has a comprehensive data quality framework. Submitting sites are required to perform monthly automated data validation checks that flag records with missing encounters or data errors so that corrections can be made; every month, each site must report that it has reviewed and resolved flagged errors.[1] A formal validation study has been conducted, in which the accuracy of 29 core variables in the BORN registry was assessed by comparing data entered into the registry with original clinical data extracted from patient health records in a sample of hospitals. The study found good agreement between the two sources, with 76% of the variables (22 of 29) having greater than 90% agreement, and 12 of the categorical elements having almost perfect (kappa 0.81–0.99) or substantial (kappa 0.61–0.80) agreement.[4]
**Canadian Census**	To obtain information on rural/urban residence and neighbourhood income fifths, we linked the study population to Statistics Canada's 2016 Census, based on postal code of maternal residence.
**Ontario Marginalization Index**	The Ontario Marginalization Index, which is derived from data from Statistics Canada's Census, quantifies the level of marginalization across the province.[5] The Index consists of four dimensions: residential instability, material deprivation, dependency, and ethnic concentration. Area-based fifths scores are available for each dimension, with the lowest fifths representing the least marginalized areas, and the highest fifths representing the most marginalized.<br><br>Residential instability identifies areas with high rates of family or housing instability. The Census indicators used to derive residential instability are: 1) percentage of population living alone; 2) percentage of population who are not youth aged 5-15 years; 3) average number of persons per dwelling; 4) percentage of dwellings that are in apartment buildings; 5) percentage of population who are

	single/divorced/widowed; 6) percentage of dwellings that are not owned; and 7) percentage of population who moved during the past five years.<br><br>Material deprivation, which is closely connected to poverty, identifies individuals and communities unable to access and attain basic material needs. The Census indicators used to derive material deprivation are: 1) percentage of population ≥20 years without a secondary diploma; 2) percentage of lone-parent families; 3) percentage of total income received from government transfer payments for population aged ≥15 years; 4) percentage of population aged ≥15 years who are unemployed; 5) percentage of population considered low-income; and 6) percentage of household dwellings in need of major repair.<br><br>Dependency identifies areas with high concentrations of residents who do not receive employment income. The Census indicators used to derive dependency are: 1) percentage of population who are aged ≥65 years; 2) dependency ratio (total population aged 0-14 years and ≥65 years / total population aged 15 to 64 years); and 3) percentage of population aged ≥15 years not participating in labour force.<br><br>Ethnic concentration identifies areas with high concentrations of recent immigrants and/or "visible minorities" (defined by Statistics Canada as "persons, other than Aboriginal peoples, who are non-Caucasian in race or non-white in colour"). Ethnic concentration is derived from two Census variables: 1) percentage of population who are recent immigrants (arrived in past five years); and 2) percentage of population who self-identify as a visible minority (Census respondents can indicate more than one race/ethnicity from a list, or can specify a group not listed on the Census questionnaire).<br><br>The Ontario Marginalization Index is linked with the BIS using postal code of maternal residence.
**COVaxON**	COVaxON, Ontario's covid-19 immunization database, contains records for all covid-19 vaccines administered in the province. Data are reported into COVaxON at the time of immunization, regardless of type of provider or delivery location (mass immunization clinic, pharmacy, etc.). Information includes vaccine product, dose number, and date(s) of vaccination. On a monthly basis, an extract of all immunization events in females aged 15-45 years is transferred to BORN Ontario, where they are deterministically linked using health card number to the BIS to identify individuals who received covid-19 vaccination during pregnancy.
**CCM**	Ontario's Case and Contact Management System (CCM) is a centralized repository for covid-19 case and contact management.[6] Each public health unit in the province collects information on covid-19-positive cases and reports it to the Ministry of Health. On a monthly basis, an extract of all PCR-confirmed positive cases in females aged 15-45 years is transferred to BORN Ontario, where they are deterministically linked using health card number with the BIS to identify individuals who had laboratory-confirmed covid-19 during pregnancy.

Supplementary Table 2. Description of study variables from the BORN Ontario birth registry

Variable	Description
Stillbirth	BORN Ontario uses the Ontario Vital Statistics Act's definition of stillbirth: "…..the complete expulsion or extraction from a person of a product of conception either after the 20th week of pregnancy or after the product of conception has attained a birth weight of 500g or more and shows no signs of life at birth." (https://www.ontario.ca/laws/statute/90v04). Stillbirth includes an antepartum or intrapartum fetal death at ≥20 weeks or ≥500 grams, with the gestational timing of the event based on the date of birth (information on the timing of fetal demise was not available).
Small for gestational age at birth	Calculated field in the birth registry based on infant sex, gestational age, and birth weight. SGA at birth is defined as a singleton live birth below the 10th centile of the sex-specific birth weight for gestational age distribution, based on a Canadian reference standard.[7]
Maternal age	Calculated field indicating maternal age at time of live birth or stillbirth.
Estimated date of conception	Best estimate of date of conception determined by ultrasound or mathematical calculation using Nägele's rule.
Parity	The number of previous live births and stillbirths (term + preterm). This is automatically calculated.
Multiple birth	Number of fetuses in the current pregnancy.
Pre-existing medical condition	Maternal health conditions and/or complications including those pre-existing, diagnosed during pregnancy or active during pregnancy. Variable was derived as a composite of the following conditions: thyroid disease, asthma, diabetes, chronic hypertension, and heart disease.
Self-reported smoking during pregnancy	Self-reported amount of smoking per day closest to time of labour/admission or at time of first prenatal visit. Smoking status includes any self-reported cigarettes that were smoked at any time during the pregnancy. This does not include marijuana or vaping.
Self-reported substance use during pregnancy	Maternal self-reported drug and substance use during pregnancy. This refers to the use of street drugs and the inappropriate use of prescription and non-prescription drugs and includes cocaine, gas/glue, hallucinogens, opioids, and cannabis. Measure of substance use included maternal self-reported cannabis exposure (i.e., smoking, vaping, consumption of edibles and cannabis products, and topical application) at any point during this pregnancy as documented in the medical record.
Pre-pregnancy maternal BMI (kg/m2)	Derived using maternal weight (kg) and height (cm). Maternal weight is reported as mother's self-reported weight closest to conception and no later than 12 weeks of gestation (metric or imperial units) and maternal height is reported in imperial or metric units.
First prenatal care visit in the first trimester	Indication that mother had a prenatal care visit with a regulated health care provider during the first trimester (<14 weeks and 0 days gestation of pregnancy) of the current pregnancy.
Birth location	The location where the birth occurred (hospital, home, other).
Healthcare provider that caught/delivered baby	Identify the health care provider who actually caught/delivered (had hands on) the baby. The language 'caught' refers to midwifery, 'delivered' refers to hospital settings. Births are considered "unattended" if the person who caught/delivered the baby is not a health care provider.

Supplementary Figure 1. Study design

A)

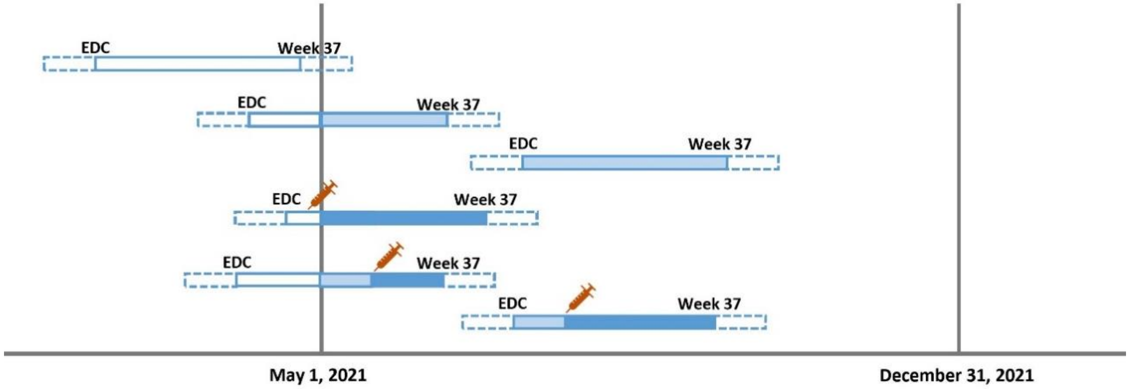

B)

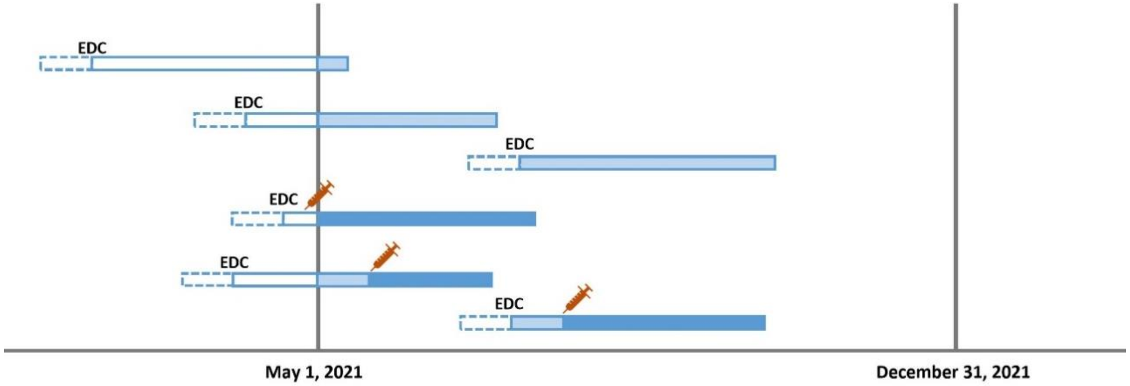

Births from May 1 to December 31, 2021 in Ontario were eligible if the last menstrual period was before March 10, 2021 (i.e., 42 weeks before the end of the study period) to prevent cohort truncation bias. Each pregnancy contributed gestational time in days starting on the estimated date of conception (pregnancy day 14); pregnancies that were ongoing on the first day of the study period contributed gestational days from May 1, 2021 (blue shading). Follow-up continued until the event occurred or until the pregnancy was censored upon reaching the end of the outcome-specific risk window (upon reaching 37 weeks' gestation for preterm birth outcome [Panel A]; upon reaching the end of the pregnancy for the small for gestational age at birth and stillbirth outcomes [Panel B]). Vaccination

status was treated as time-varying during the risk window—light blue shading depicts unvaccinated gestational time in the risk window and dark blue shading depicts vaccinated gestational time during the risk window.

Supplementary Table 3. Subgroup analyses

**Outcome**	**Adjusted hazard ratio (95% CI)** [a]
Preterm birth <37 weeks [b, c]	
Original results shown in manuscript Table 4	1.02 (0.96, 1.08)
Original model using multivariable adjustment	0.98 (0.93, 1.04)
No covid-19 vaccination	1.00
Trimester of dose 1	
1st trimester	0.98 (0.87, 1.10)
2nd trimester	0.98 (0.91, 1.04)
3rd trimester	1.00 (0.92, 1.08)
Vaccine product for dose 1 [d]	
BNT162b2	0.98 (0.93, 1.04)
mRNA-1273	1.00 (0.91, 1.09)
Number of doses [e]	
1 dose	1.04 (0.97, 1.12)
2 doses	0.94 (0.88, 1.01)
mRNA vaccine product for dose 1 and 2 [d, f]	
BNT162b2 + BNT162b2	1.00 (0.93, 1.08)
mRNA-1273 + mRNA-1273	1.03 (0.92, 1.16)
BNT162b2 + mRNA-1273 / mRNA-1273 + BNT162b2	1.04 (0.90, 1.19)
Small for gestational age (SGA) at birth [g, h,]	
Original results shown in manuscript Table 4	0.98 (0.93, 1.03)
Original model using multivariable adjustment	0.98 (0.93, 1.03)
No covid-19 vaccination	1.00

Trimester of dose 1	
1st trimester	1.00 (0.90, 1.12)
2nd trimester	1.02 (0.96, 1.08)
3rd trimester	0.93 (0.88, 1.00)
Vaccine product for dose 1 [d]	
BNT162b2	0.98 (0.93, 1.03)
mRNA-1273	0.97 (0.89, 1.05)
Number of doses [e]	
1 dose	1.09 (1.01, 1.16)
2 doses	0.92 (0.87, 0.97)
mRNA vaccine product for dose 1 and 2 [d, f]	
BNT162b2 + BNT162b2	0.97 (0.92, 1.04)
mRNA-1273 + mRNA-1273	0.92 (0.83, 1.03)
BNT162b2 + mRNA-1273 / mRNA-1273 + BNT162b2	0.85 (0.75, 0.96)

[a] Conventional multivariable adjustment was used for subgroup analyses instead of using stabilized inverse probability of treatment weights, since the weights were derived from a propensity score model predicting vaccination during pregnancy vs. no vaccination during pregnancy, whereas the subgroup analyses use alternate exposure definitions. The proportional hazards assumption for all time-fixed covariates was assessed using the first imputed dataset and found to be fulfilled based on examination of Schoenfeld residual plots. Multivariable models were performed on five multiple imputation datasets and adjusted for week of conception, maternal age, nulliparity, multifetal pregnancy, pre-existing maternal medical condition (composite of: asthma, chronic hypertension, diabetes, heart disease, thyroid disease), first prenatal care visit in the first trimester, smoking during pregnancy, self-reported substance use during pregnancy, maternal body mass index ≥30 kg/m^2, neighbourhood median family income fifths, rural residence, public health region of residence, and four marginalization indices (residential instability, material deprivation, dependency, ethnic concentration).
[b] Risk window for preterm birth: 36 weeks+6 days of gestation (pregnancy day 258). Vaccination status was included as a time-varying exposure.
[c] Among live births only.
[d] Analysis excluded non-mRNA (e.g., viral vector-based) covid-19 vaccines.
[e] Doses had to occur in the outcome-specific risk window to be counted and both doses were handled as time-varying exposures.
[f] Analysis was limited to individuals who had received both vaccine doses in the outcome-specific risk window.
[g] Risk window for SGA at birth: end of pregnancy. Vaccination status was included as a time-varying exposure.
[h] Among singleton live births only. Records with a gestational age either below or above the values provided in the reference standard used to classify SGA at birth, or with missing information on infant sex and/or birth weight were excluded from the analyses (see table 4 footnote in main text).

Supplementary Table 4. Sensitivity analyses

**Outcome**	**Adjusted hazard ratio [a,b] (95% CI)**
Preterm birth <37 weeks [c]	
Original results shown in manuscript Table 4	1.02 (0.96, 1.08)
Original model using multivariable adjustment	0.98 (0.93, 1.04)
Covid-19 included as a time-varying variable	0.99 (0.93, 1.04)
Excluding those who had covid-19 during pregnancy [d]	0.99 (0.93, 1.04)
Stratified by neighbourhood income fifths	
Fifths 4 and 5 (highest)	0.96 (0.88, 1.05)
Fifths 1, 2, and 3 (lowest)	1.00 (0.94, 1.08)
Unvaccinated group limited to those vaccinated after pregnancy	0.94 (0.88, 1.00)
Among singleton births only	1.00 (0.94, 1.06)
Spontaneous preterm birth <37 weeks [c, e]	
Original results shown in manuscript Table 4	0.96 (0.90, 1.03)
Original model using multivariable adjustment	0.93 (0.87, 1.00)
Covid-19 included as a time-varying variable	0.93 (0.87, 1.00)
Excluding those who had covid-19 during pregnancy [d]	0.93 (0.87, 1.00)
Stratified by neighbourhood income fifths	
Fifths 4 and 5 (highest)	0.94 (0.84, 1.06)
Fifths 1, 2, and 3 (lowest)	0.93 (0.85, 1.02)
Unvaccinated group limited to those vaccinated after pregnancy	0.89 (0.82, 0.97)
Among singleton births only	0.95 (0.88, 1.03)

Very preterm birth <32 weeks [c]	
Original results shown in manuscript Table 4	0.80 (0.67, 0.95)
Original model using multivariable adjustment	0.81 (0.68, 0.96)
Covid-19 included as a time-varying variable	0.81 (0.68, 0.96)
Excluding those who had covid-19 during pregnancy [d]	0.83 (0.70, 0.98)
Stratified by neighbourhood income fifths	
Fifths 4 and 5 (highest)	0.61 (0.45, 0.83)
Fifths 1, 2, and 3 (lowest)	0.93 (0.76, 1.14)
Unvaccinated group limited to those vaccinated after pregnancy	0.76 (0.62, 0.92)
Among singleton births only	0.81 (0.67, 0.98)
Small for gestational age (SGA) at birth [f]	
Original results shown in manuscript Table 4	0.98 (0.93, 1.03)
Original model using multivariable adjustment	0.98 (0.93, 1.03)
Covid-19 included as a time-varying variable	0.98 (0.94, 1.03)
Excluding those who had covid-19 during pregnancy [d]	1.04 (1.00, 1.10)
Stratified by neighbourhood income fifths	
Fifths 4 and 5 (highest)	1.01 (0.93, 1.09)
Fifths 1, 2, and 3 (lowest)	1.06 (1.00, 1.13)
Unvaccinated group limited to those vaccinated after pregnancy	0.98 (0.93, 1.04)
Stillbirth	

Original results shown in manuscript Table 4	0.65 (0.51, 0.84)
Original model using multivariable adjustment	0.65 (0.51, 0.83)
Covid-19 included as a time-varying variable	0.65 (0.51, 0.83)
Excluding those who had covid-19 during pregnancy [d]	0.64 (0.50, 0.82)
Stratified by neighbourhood income fifths	
Fifths 4 and 5 (highest)	0.90 (0.61, 1.34)
Fifths 1, 2, and 3 (lowest)	0.52 (0.37, 0.73)
Unvaccinated group limited to those vaccinated after pregnancy	0.71 (0.52, 0.96)
Among singleton births only	0.74 (0.57, 0.96)
Individuals with dose 1 administered within 7 days of delivery reclassified as unvaccinated	0.62 (0.48, 0.80)

[a] Conventional multivariable adjustment was used for sensitivity analyses instead of using stabilized inverse probability of treatment weights, since the weights were derived from a propensity score model predicting vaccination during pregnancy vs. no vaccination during pregnancy in the full study population, whereas several sensitivity analyses used subgroups of the population in stratified analyses. The proportional hazards assumption for all time-fixed covariates was assessed using the first imputed dataset and found to be fulfilled based on examination of Schoenfeld residual plots. Models were performed on five multiple imputation datasets and adjusted for week of conception, maternal age, nulliparity, multifetal pregnancy, pre-existing maternal medical condition (composite of: asthma, chronic hypertension, diabetes, heart disease, thyroid disease), first prenatal care visit in the first trimester, smoking during pregnancy, self-reported substance use during pregnancy, maternal body mass index ≥ 30 kg/m^2, neighbourhood median family income fifths, rural residence, public health region of residence, and four marginalization indices (residential instability, material deprivation, dependency, ethnic concentration).
[b] Vaccination was treated as a time-varying exposure within outcome-specific risk windows.
[c] Among live births only.
[d] Limited to those who did not have covid-19 within the outcome-specific risk window.
[e] For spontaneous preterm birth, medically-initiated preterm births were censored at delivery.[8]
[f] Among singleton live births only. Dates of vaccine doses were lagged by 14 days since SGA at birth cannot be associated with a proximal exposure. Records with a gestational age either below or above the values provided in the reference standard used to classify SGA at birth, or with missing information on infant sex and/or birth weight were excluded from the analyses (see table 4 footnote in main text).

References

1. Murphy MSQ, Fell DB, Sprague AE, et al. Data Resource Profile: Better Outcomes Registry & Network (BORN) Ontario. *Int J Epidemiol* 2021;50:1416-7h.
2. Association of Ontario Midwives. Birthplace Options - Why Give Birth at Home? Accessed June 24, 2022. https://www.ontariomidwives.ca/home-birth.
3. Huang T, Dougan S, Walker M, Armour CM, Okun N. Trends in the use of prenatal testing services for fetal aneuploidy in Ontario: a descriptive study. *CMAJ Open* 2018;6:E436-E444.
4. Dunn S, Lanes A, Sprague AE, et al. Data accuracy in the Ontario birth Registry: a chart re-abstraction study. *BMC Health Serv Res* 2019;19:1001.
5. Public Health Ontario. Ontario Marginalization Index (ON-Marg). Accessed June 24, 2022. https://www.publichealthontario.ca/en/data-and-analysis/health-equity/ontario-marginalization-index.
6. Ontario Ministry of Health. Protecting Ontarians through enhanced case and contact management. Accessed June 24, 2022. https://www.ontario.ca/page/case-and-contact-management-strategy.
7. Kramer MS, Platt RW, Wen SW, et al. A new and improved population-based Canadian reference for birth weight for gestational age. *Pediatrics* 2001;108:E35.
8. Savitz DA, Dole N, Herring AH, et al. Should spontaneous and medically indicated preterm births be separated for studying aetiology? *Paediatr Perinat Epidemiol* 2005;19:97-105.
